# Cell-permeable succinate prodrugs bypass mitochondrial complex I deficiency

**DOI:** 10.1038/ncomms12317

**Published:** 2016-08-09

**Authors:** Johannes K. Ehinger, Sarah Piel, Rhonan Ford, Michael Karlsson, Fredrik Sjövall, Eleonor Åsander Frostner, Saori Morota, Robert W. Taylor, Doug M. Turnbull, Clive Cornell, Steven J. Moss, Carsten Metzsch, Magnus J. Hansson, Hans Fliri, Eskil Elmér

**Affiliations:** 1 Mitochondrial Medicine, Department of Clinical Sciences Lund, Faculty of Medicine, Lund University, BMC A13, 221 84 Lund, Sweden; 2 NeuroVive Pharmaceutical AB, Medicon Village, 223 81 Lund, Sweden; 3 Department of Otorhinolaryngology, Head and Neck Surgery, Department of Clinical Sciences Lund, Lund University, Skåne University Hospital, 221 85 Lund, Sweden; 4 Selcia Ltd, Fyfield Business and Research Park, Fyfield Road, Ongar CM5 0GS, Essex, UK; 5 Department of Intensive Care and Perioperative Medicine, Skåne University Hospital, 205 02 Malmö, Sweden; 6 Wellcome Trust Centre for Mitochondrial Research, Institute of Neuroscience, The Medical School, Newcastle University, Newcastle upon Tyne NE2 4HH, UK; 7 Isomerase Therapeutics Ltd, Chesterford Research Park, Cambridge CB10 1XL, UK; 8 Anaesthesiology and Intensive Care, Department of Clinical Sciences Lund, Faculty of Medicine, Lund University, 221 85 Lund, Sweden; 9 Mitopharm Ltd, Fyfield Business and Research Park, Fyfield Road, Ongar CM5 0GS, Essex, UK; 10 Clinical Neurophysiology, Department of Clinical Sciences Lund, Lund University, Skåne University Hospital, 221 85 Lund, Sweden

## Abstract

Mitochondrial complex I (CI) deficiency is the most prevalent defect in the respiratory chain in paediatric mitochondrial disease. This heterogeneous group of diseases includes serious or fatal neurological presentations such as Leigh syndrome and there are very limited evidence-based treatment options available. Here we describe that cell membrane-permeable prodrugs of the complex II substrate succinate increase ATP-linked mitochondrial respiration in CI-deficient human blood cells, fibroblasts and heart fibres. Lactate accumulation in platelets due to rotenone-induced CI inhibition is reversed and rotenone-induced increase in lactate:pyruvate ratio in white blood cells is alleviated. Metabolomic analyses demonstrate delivery and metabolism of [^13^C]succinate. In Leigh syndrome patient fibroblasts, with a recessive *NDUFS2* mutation, respiration and spare respiratory capacity are increased by prodrug administration. We conclude that prodrug-delivered succinate bypasses CI and supports electron transport, membrane potential and ATP production. This strategy offers a potential future therapy for metabolic decompensation due to mitochondrial CI dysfunction.

## Abstract (web summary)

Mitochondrial complex I deficiency is the most common respiratory chain defect in mitochondrial disease in children and currently there is no effective treatment. In this study, the authors show that succinate prodrugs can alleviate metabolic decompensation in Leigh syndrome patient fibroblasts.

## Graphical abstract

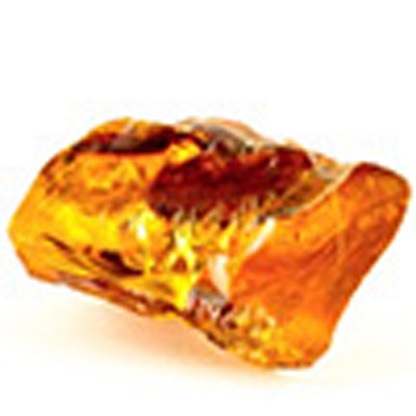

Paediatric mitochondrial disease due to complex I (CI) deficiency is a heterogeneous group of disorders, and can be due to alterations in either the nuclear or mitochondrial genome. It is the most prevalent defect in the respiratory chain in paediatric patients and often leads to serious or fatal neurological presentations, such as Leigh syndrome^1^. There are currently very limited evidence-based treatment options directed towards mitochondrial respiratory chain dysfunction^2^,^3^. Succinate is a mitochondrial substrate metabolized through complex II (CII). It is not cell membrane-permeable and exogenously given succinate has limited uptake into cells.

Here we describe that cell membrane-permeable prodrugs of succinate provide increased ATP-linked mitochondrial oxygen consumption in CI-deficient human cells and tissues, which offers a potential future intervention for patients with metabolic decompensation due to mitochondrial CI dysfunction.

## Results

### Drug development and screening

**Figure 1:**
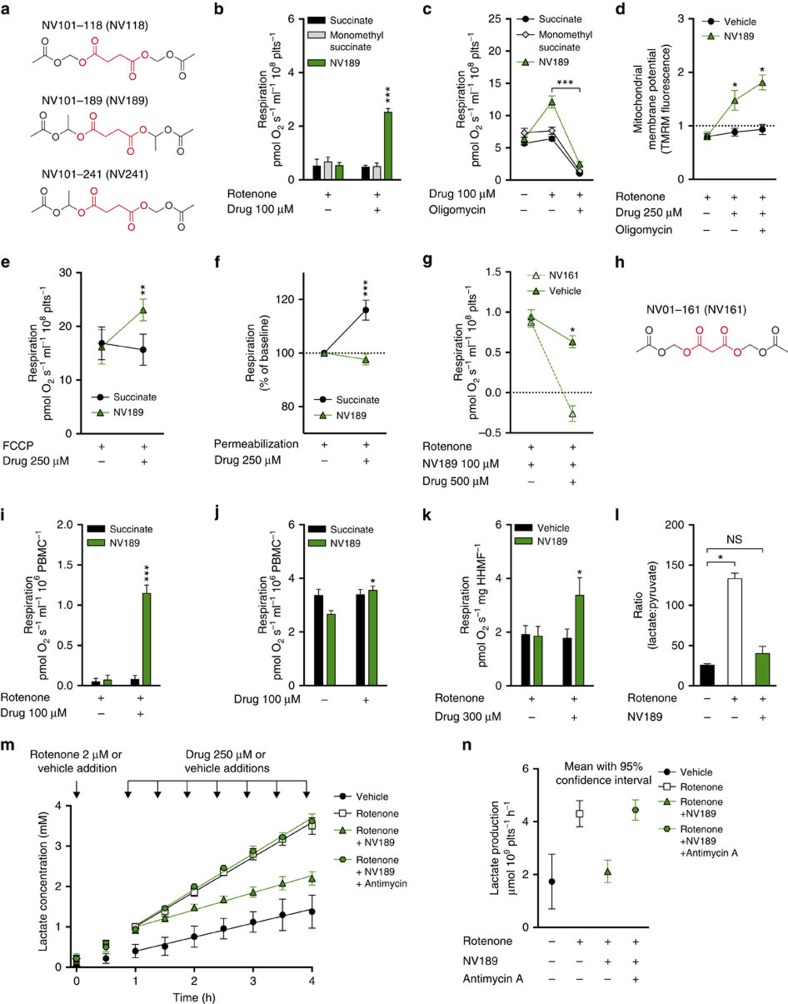
Effects of mitochondrial complex II stimulation by the succinate prodrug NV189. (**a**) Structures of NV118, NV189 and NV241, succinate highlighted in red. (**b**) Respiration in platelets (plts) with rotenone-induced mitochondrial complex I (CI) inhibition. (**c**) ATP-generating respiration in platelets. (**d**) Mitochondrial membrane potential in complex I-inhibited platelets, ratio of basal TMRM fluorescence, *n*=4. (**e**) Respiration in platelets with FCCP-induced uncoupling. (**f**) Respiration in digitonin-permeabilized platelets. (**g**) Effect on respiration in platelets with addition of the cell-permeable complex II inhibitor NV161, * indicate significant difference between NV161 and vehicle, *n*=4. (**h**) Structure of NV161, malonate highlighted in red. (**i**) Respiration in peripheral blood mononuclear cells (PBMCs) with rotenone-induced CI inhibition, *n*=4. (**j**) Convergent respiration in PBMCs, *n*=4, * indicate significant difference between pre and post dosing. (**k**) Respiration in human heart muscle fibres (HHMFs), *n*=5. (**l**) Lactate:pyruvate ratio in PBMCs at baseline, after rotenone CI inhibition and after treatment with NV189, *n*=4. * indicates significant difference using Friedmans non-parametric paired test with Dunn's multiple comparisons test of all groups against control. For three data points, pyruvate was below detection limit and the estimated lower-quantification limit was used for calculating the ratio. (**m**) Lactate accumulation in 2 ml buffer containing 400 × 10^6^ platelets, incubated with or without rotenone, antimycin A and NV189, *n*=5. (**n**) Lactate production in platelets, data quantification from previous panel. Mean with 95% confidence interval. All respirometric experiments in human platelets were performed with *n*=6 individuals donors if not otherwise stated. All data presented as mean and s.e. if not otherwise stated. In all experiments, blood cells from separate donors are used for each *n*. *P<0.05, **P<0.01, ***P<0.001 (two-tailed paired or unpaired Student's *t*-test as appropriate, difference between test compound and control if not otherwise stated).

In a drug discovery program, >50 different prodrugs of succinate^4^ were designed, synthesized and evaluated for cell permeability and ability to support respiration independent of CI in human peripheral blood cells from healthy donors (platelets and mononuclear cells (PBMCs)) using an Oroboros O2k respirometer. Three compounds were selected for further evaluation: NV101-118 (NV118, diacetoxymethyl succinate), NV101-189 (NV189, bis-(1-acetoxy-ethyl) succinate) and NV101-241 (NV241, 1-acetoxyethyl acetoxymethyl succinate) (Fig. 1a). This article focuses on NV189, but qualitatively the results for all three prodrugs were similar and data on the other compounds are presented as Supplementary Figs.

### Increased CII-linked respiration

At 100 μM, NV189 increased mitochondrial oxygen consumption in intact platelets with CI inhibition induced by the mitochondrial toxin rotenone (2 μM). Neither succinate nor monomethyl succinate, a monoester of succinate previously reported to be cell permeable^5^, increased mitochondrial respiration (Fig. 1b; Supplementary Fig. 1a). In cells with normal CI function, oxygen consumption was also increased upon addition of 100 μM NV189 (Fig. 1c; Supplementary Fig. 1b). To exclude the possibility that increased respiration was due to an induction of proton leak through the mitochondrial inner membrane (uncoupling), the platelets were treated with the ATP synthase inhibitor oligomycin. This prompted a significant decrease in oxygen consumption, which indicates the extent of respiration linked to ADP phosphorylation (Fig. 1c; Supplementary Fig. 1b). Increased substrate supply, rather than uncoupling, was further demonstrated by measuring mitochondrial inner membrane potential with the positively charged membrane-permeable probe tetramethylrhodamine methyl ester (TMRM) in non-quench mode using fluorescence-activated cell sorting. TMRM fluorescence was increased in CI-inhibited human platelets upon addition of 250 μM NV189 and fluorescence increased further with ATP synthase inhibition, indicating mitochondrial membrane hyperpolarization (Fig. 1d). Cells with maximal uncoupled respiratory chain activity via titration of the protonophore carbonyl cyanide *p*-(trifluoromethoxy) phenylhydrazone (FCCP) increased oxygen consumption even more with addition of 250 μM NV189, further indicating increased substrate supply to the respiratory chain (Fig. 1e; Supplementary Fig. 1c). In blood cells, pre-permeabilized with the detergent digitonin, 250 μM NV189 did not induce any increase in respiration, while succinate control did, showing the need for intracellular metabolism for succinate to be released and made available to the mitochondria (Fig. 1f; Supplementary Fig. 1d). To confirm that the increase in respiration is specifically due to respiration through CII, a cell-permeable prodrug of the CII inhibitor malonate, NV01-161, (NV161, diacetoxymethyl malonate, Fig. 1h) was designed, synthesized and evaluated (Supplementary Fig. 2). Intact cells exposed to succinate prodrugs were treated with NV161 with ensuing decrease in respiration (Fig. 1g; Supplementary Fig. 1e). The applicability of the platelet data to other cell types was evaluated by assessing respiration in PBMCs treated with NV189 with or without CI inhibition with similar results to those in platelets (Fig. 1i,j; Supplementary Fig. 1f,g).

Paediatric mitochondrial diseases primarily display symptoms from energy intense organs such as the liver, brain, muscles, retina and cochlea. In some reports, 30–40% of paediatric patients with respiratory chain CI dysfunction present with cardiomyopathy^6^,^7^, a condition that is linked to higher mortality^8^. Human atrial heart muscle biopsies from elective surgery were acquired and the fibres gently separated using forceps. The fibres were incubated with the CI inhibitor rotenone and subsequently treated with succinate prodrug, eliciting an increase in oxygen consumption (Fig. 1k; Supplementary Fig. 1h).

### Attenuated lactate production

A hallmark of mitochondrial disease is lactic acidosis. When the mitochondrial energy production fails to comply with demand, pyruvate is converted to lactate to maintain the NAD^+^ pool, causing increased lactate levels and decreased pH in blood and cerebrospinal fluid in the patients. About 80% of patients with mitochondrial disease show signs of lactate accumulation^6^,^8^,^9^. We incubated human platelets with or without 2 μM rotenone and measured lactate accumulation in media over time. With CI inhibition, the cells displayed a significantly higher lactate production than control, 4.30±0.24 μmol lactate per 10^9^ cells per hour compared with control level 1.73±0.5 (regression slope±s.d.), but with incubation with NV189 the rotenone-induced lactate production was similar to control level (1.26±0.19). To verify the viability of the cell preparation, the glycolytic pathway upon drug addition and the specificity of CII-mediated ATP supply, cells were incubated with NV189, rotenone and an inhibitor of the downstream respiratory chain complex III (antimycin A, 1 μg ml^−1^), eliciting lactate production at the level of that of rotenone alone (4.44±0.19; Fig. 1m,n; Supplementary Fig. 1i).

### Metabolomics confirms metabolism of delivered succinate

**Figure 2:**
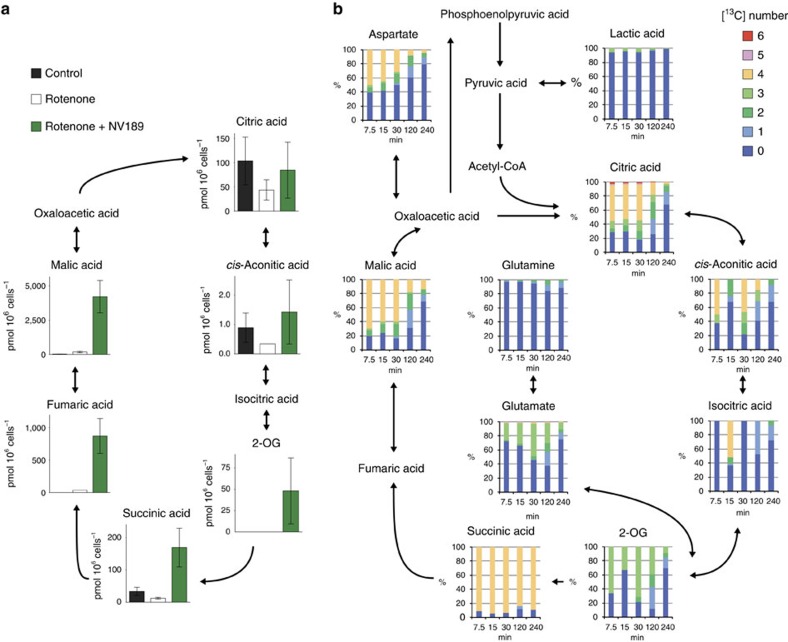
Intracellular metabolism of exogenous prodrug-delivered succinate. (**a**) TCA cycle intermediates in peripheral blood mononuclear cells after 20 min incubation with or without rotenone and NV189 quantified using capillary electrophoresis mass spectrometry, *n*=4. Data presented as mean and s.d. (**b**) Fraction of [^13^C] isotope labelled carbons in TCA cycle intermediates and related metabolites in human platelets incubated with [1, 2, 3, 4-^13^C_4_]NV118 for 7.5, 15, 30, 120 or 240 min. Mean of *n*=2. 2-OG, 2-oxoglutaric acid.

To elucidate the intracellular metabolism of NV189, a metabolomic assay was performed on PBMCs from four healthy donors. Cells were incubated with or without rotenone and with or without NV189 for 20 min. Using quantitative capillary electrophoresis mass spectrometry (CE-MS), the concentrations of 116 metabolites were determined. Delivery of intracellular succinate and anaplerosis of tricarboxylic acid (TCA) cycle intermediates were confirmed (Fig. 2a; Supplementary Fig. 3). The lactate:pyruvate ratio was increased when cells were inhibited with rotenone and normalized when the cells were treated with NV189 (Fig. 1l). No conclusive alterations due to drug treatment in metabolism of succinyl-CoA-related amino acids or glycolysis could be shown. Levels of cysteine were decreased, which could indicate oxidative stress. To investigate the time course of intracellular metabolism of delivered succinate, [1, 2, 3, 4-^13^C_4_]NV118 was synthesized, whereby the carbon atoms in NV118 that upon release would comprise the four carbon atoms in succinate were enriched with the stable isotope ^13^C. This distinguishes between endogenous TCA cycle intermediates and metabolites originating from the prodrug-delivered succinate. NV118 rather than NV189 was used due to relatively less complex synthesis. Human platelets were then incubated with [1, 2, 3, 4-^13^C_4_]NV118 for 7.5, 15, 30, 120 and 240 min. Even at the first time point, [^13^C_4_]malate and [^13^C_4_]citrate were observed, demonstrating rapid entry of [^13^C_4_]succinate into the TCA cycle (Fig. 2b). There was also [^13^C_6_]citrate present, which indicates that [^13^C]oxaloacetate or [^13^C]malate had converted to pyruvate and through acetyl-CoA formed citrate with [^13^C_4_]oxaloacetate (Fig. 2b; Supplementary Fig. 4), demonstrating continuous metabolism in the TCA cycle. The ratio of labelled species gradually declined with time but still after 240 min, there was a supply of labelled succinate available.

### Respiration increased in Leigh syndrome patient fibroblasts

**Figure 3:**
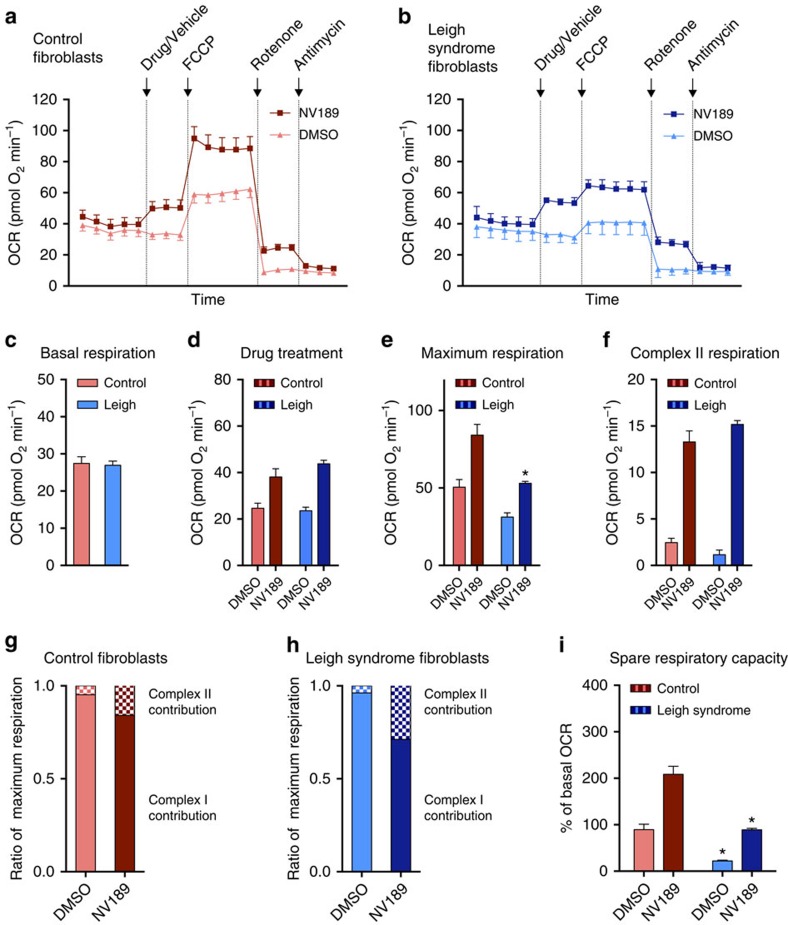
Succinate prodrug treatment of mitochondrial complex I-deficient Leigh syndrome patient fibroblasts. (**a**,**b**) Oxygen consumption rate (OCR) in three control fibroblast cell lines and a mitochondrial complex I-deficient cell line (recessive *NDUFS2* mutation) treated with NV189 or vehicle. (**c**–**f**) Quantification of OCR in control and patient fibroblasts for each respiratory state. (**g**,**h**) Relative contribution of complex I- and complex II-linked respiration to maximum uncoupled respiration in patient cells and control cell lines. (**i**) Spare respiratory capacity, defined as per cent increase from endogenous baseline to maximum uncoupled respiration. Data presented as mean and s.e. of *n*=3 experiments from separate cell culture flasks performed with eight technical replicates each time for each cell lines. Data from the three control cell lines are pooled. *P<0.05 (two-tailed unpaired Student's *t*-test, difference between Leigh and control cell lines).

To evaluate the effect of NV189 on patient cells, fibroblasts from a patient with Leigh syndrome due to recessive nuclear DNA mutations in the structural CI gene *NDUFS2* and three control cell lines were investigated using a Seahorse Bioscience XF^e^ 96 Extracellular Flux Analyzer (Fig. 3; Supplementary Fig. 5). The patient fibroblasts have previously been shown to exhibit severely decreased activity of CI, decreased CI assembly and lower expression of CI structural proteins^10^. Pooled data from all experiments (Fig. 3c,d; Supplementary Fig. 5c,d) revealed a 25% decrease in basal oxygen consumption rate (OCR) and a 42% reduction in maximum uncoupled respiration in the Leigh syndrome patient cells compared with the mean of the control cell lines. After addition of NV189, the OCR was similar between patient and controls (Fig. 3d; Supplementary Fig. 5d). The patient cells had lower maximum respiration compared with control cells, but in the presence of NV189 the OCR of patient cells was similar to that of untreated control cells (Fig. 3e and Supplementary Fig. 5e). After rotenone inhibition of CI, both cell types elicited clear remaining respiratory activity in cells treated with NV189 (Fig. 3f; Supplementary Fig. 5f). The relative contribution of flux through CII to maximum uncoupled respiration for NV189 was 4.8% in the control cell lines and 3.8% in the Leigh syndrome cells. With treatment, this increased to 15.9% in control cells and to 28.8% in patient cell (Fig. 3g,h; Supplementary Fig. 5g,h), illustrating the dependence of CII substrates in the patient cells to reach normal respiratory function. When patient cells were treated with the prodrugs, the spare respiratory capacity (respiratory reserve, the ability of the cells to increase respiration from the endogenous baseline) as percentage of the endogenous baseline was similar to that of the control cell lines (Fig. 3i; Supplementary Fig. 5i). Succinate or dimethyl succinate (an ester previously suggested to be cell permeable^11^,^12^) did not exert any effects on either cell type (Supplementary Fig. 6).

## Discussion

**Figure 4:**
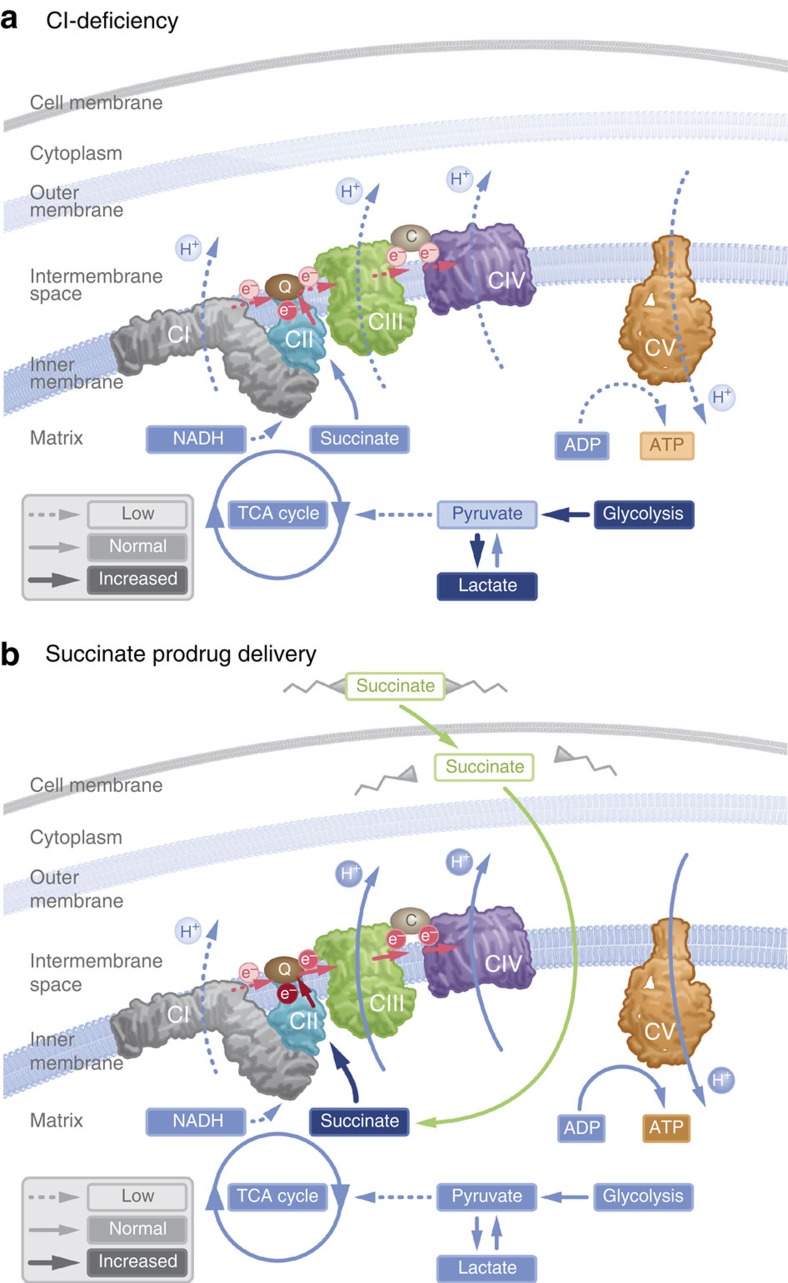
Delivery of succinate to the intracellular space via a prodrug strategy. (**a**) Dysfunction in mitochondrial complex I reduces electron flow through the respiratory chain, shift metabolism towards glycolysis, induce lactate accumulation and limit ATP production. (**b**) Cell membrane-permeable prodrugs of succinate access the intracellular space and release succinate, enabling increased electron transport, respiration and ATP production through complex II, thus bypassing the deficiency in mitochondrial complex I.

Mitochondrial disorders frequently present early in life with failure to thrive, myopathy and neuropathy, but the symptoms are very diverse^13^. At least 1 in 8,000 births will develop a mitochondrial disease^14^. Mitochondrial diseases are usually progressive and have a fluctuating clinical course. Periods of deterioration, such as during an intercurrent viral infection, are prompted by the increase in metabolic demand that the mitochondria cannot compensate for, resulting in metabolic decompensation^15^. It is an area of large unmet medical need as few evidence-based treatment options are available^2^. We describe here three model compounds of the first generation of a new pharmacological strategy to metabolically support these patients during time of metabolic decompensation. The current compounds lack sufficient plasma stability to be suitable for *in vivo* use. A cell-permeable prodrug of succinate can enter the cell independent of active uptake and subsequently release succinate. By supplying the mitochondria with substrates for CII, cells that are unable to comply with metabolic demand due to limitations at CI, or upstream thereof, may increase ATP production through oxidative phosphorylation, demonstrated here by the normalization of spare respiratory capacity in metabolically defect patient cells (Fig. 3i). By supporting aerobic metabolism, the relative dependence on glycolysis for ATP generation is alleviated and lactate production is attenuated (Figs 1n and 4). Utilizing a cell-permeable prodrug strategy to deliver a TCA cycle intermediate to the intracellular space is a feasible pharmacologic strategy with potential benefit in conditions affecting mitochondrial function, such as CI dysfunction or TCA cycle intermediate depletion in organic acidemias. Here we demonstrate that prodrug-delivered succinate can alleviate metabolic decompensation due to CI-related mitochondrial dysfunction.

## Methods

### Human peripheral blood cells

The blood cell protocols were approved by the regional ethics committee of Lund University, Sweden (permit no. 2013/181), and written informed consent was acquired from each participant. From healthy volunteers, venous blood was drawn to K_2_EDTA tubes (Vacutainer, BD, Franklin Lakes, USA) via venous puncture. Platelets were isolated with consecutive centrifugation steps as previously described^16^. Peripheral blood mononuclear cells (PBMCs) were isolated using Lymphoprep (Axis-Shield, Dundee, Scotland). Erythrocytes and PBMCs were loosely pelleted by 10 min centrifugation at 500*g*. The pellet was resuspended in saline, layered on a Ficoll gradient and centrifuged at 800*g* for 20–30 min. The resulting leukocyte layer was collected, resuspended in saline and pelleted by 5 min centrifugation at 250*g*. The supernatant was removed and the pellet resuspended in 100–200 μl of saline. Blood cells were counted using an automated hematocytometer (SweLab Alfa, Boule Diagnostics, Sweden). The number of biological replicates (blood cells derived from different individual donors) are provided in the respective figure legends for all experiments.

### Human cardiac muscle samples

Biopsies of human cardiac muscle were obtained at the Department of Cardiothoracic Surgery, Skåne University Hospital, Lund, Sweden. Pre-surgery informed consent was obtained from patients undergoing planned open-heart surgery such as mitral valve repair or maze procedure for treatment of atrial fibrillation. Only superfluous tissue that otherwise would have been discarded or located behind the suture line for the cannulation catheter was collected (up to 2 g was collected, 50–100 mg used for each experiment). Ethical permission was granted by the regional ethical review board of Lund, Sweden (permit no. 2013/271, 2013/701). The biopsy was immediately transferred to ice-cold preservation solution (BIOPS; 10 mM Ca-EGTA buffer, 0.1 μM free calcium, 20 mM imidazole, 20 mM taurine, 50 mM K-MES, 0.5 mM dithiothreitol, 6.56 mM MgCl_2_, 5.77 mM ATP and 15 mM phosphocreatine, pH 7.1). It was thereafter dissected under microscope using forceps to gently separate the fibres and remove any fat and connective tissue. Biopsy wet weight was obtained before respiratory measurements (Precisa 40SM-200A, Abbot, USA).

### Cultured fibroblasts

Permit for research on fibroblasts was granted by the Newcastle and North Tyneside 1 NRES Committee (REC reference 2002/205). A cultured skin fibroblast cell line from a patient with clinical Leigh syndrome due to a deficiency in the nuclear encoded structural mitochondrial CI protein *NDUFS2* (p.Arg118Gln; p.Met292Thr mutations), and relevant control cell lines from healthy donors were provided by the Wellcome Trust Centre for Mitochondrial Research at Newcastle University, UK^10^. The fibroblasts were cultured in minimum essential medium (MEM) supplemented with 10% fetal bovine serum, 1% MEM vitamins, 1% MEM non-essential amino acids, 2 mM L-glutamine, 50 μg ml^−1^ streptomycin, 50 U ml^−1^ penicillin, 50 μg ml^−1^ uridine and 1 mM sodium pyruvate at 37 °C and 5% CO_2_. Cells were collected using trypsin and split or used for analysis at ∼70–80% confluence and counted using an automated cell counter (TC20, Bio-Rad, Hercules, USA).

### Respirometry

For cells in monolayers, the Seahorse Bioscience XF^e^ 96 Extracellular Flux Analyser (Seahorse Bioscience, North Billerica, USA) was the instrument of choice, and for cells in suspension such as blood cells the Oroboros O2k (Oroboros Instruments, Innsbruck, Austria) was used. Respiratory measurements using Oroboros O2k were performed in stirred (750 r.p.m.) 2 ml glass chambers at 37 °C. The media MiR05 (sucrose 110 mM, HEPES 20 mM, taurine 20 mM, K-lactobionate 60 mM, MgCl_2_ 3 mM, KH_2_PO_4_ 10 mM, EGTA 0.5 mM and bovine serum albumin 1 g l^−1^, pH 7.1) was used in all experiments^16^,^17^. Data were recorded using the DatLab software version 4, 5 or 6 (Oroboros Instruments). Correction for instrumental background and air calibration was performed according to the manufacturer's instructions.

All experiments with platelets were performed with cell concentrations of 200 × 10^6^ cells per ml and all experiments with PBMCs with 5 × 10^6^ cells per ml. In experiments with human heart fibres, ∼10 mg of tissue was used in each run. To inhibit mitochondrial CI, rotenone (2 μM) was used and to inhibit mitochondrial complex III, antimycin A (1 μg ml^−1^) was used. ATP synthase was inhibited using oligomycin (1 μg ml^−1^), evaluating the contribution of respiration independent of ADP phosphorylation. Maximum uncoupled respiration of the electron transport system was induced by titration of the protonophore carbonyl cyanide FCCP until no further increase in respiration was detected. The test compound or control substances (succinate, dimethyl succinate, monomethyl succinate, malonate, dimethyl malonate or dimethylsulphoxide (DMSO)) were dosed as indicated in each figure.

Respirometric measurements in fibroblasts were performed using a Seahorse Bioscience XF^e^ 96 Extracellular Flux Analyzer. The day before the experiment, fibroblasts were seeded out at 25,000 cells per well in cell growth medium in collagen-coated 96-well plates and kept at 37 °C and 5% CO_2_ overnight. Before the experiment, the growth medium was replaced by XF-Base Medium containing 2 mM L-glutamine, 5 mM sodium pyruvate and 10 mM glucose (pH 7.4) and the cells were kept at 37 °C 1 h at atmospheric O_2_ and CO_2_. Oxygen consumption was measured at routine state and after addition of 500 μM of NV241 or NV189, its vehicle DMSO, dimethyl succinate or disodium succinate, followed by different concentrations of FCCP (0.125, 0.5, 1.0 and 1.5 μM), 2 μM rotenone and 1 μg ml^−1^ antimycin A. After FCCP and drug addition, the first data point was generally used, if not another data point was clearly higher, and for the remaining states the last data point before the subsequent addition was used. The FCCP dosing resulting in the highest uncoupled respiration was chosen for analysis for each experiment with each cell line and treatment.

All respirometric measurements, with the exception of the human heart fibre data, were corrected for non-mitochondrial oxygen consumption, obtained after the addition of antimycin A.

### Lactate

Platelets (*n*=5 individual donors, 200 × 10^6^ cells per ml) were incubated in PBS for 4 h with rotenone (2 μM), rotenone and antimycin A (1 μg ml^−1^) combined or the vehicle for rotenone (DMSO). At *t*=60 min, additions of 250 μM NV118, NV189, NV241 or vehicle (DMSO) were initiated and repeated every 30 min throughout the experiment. Lactate levels were determined every 30 min using a Lactate ProTM 2 blood lactate meter (Arkray, Alere AB, Lidingö, Sweden)^18^. Incubation was performed at 37 °C at a stirrer speed of 750 r.p.m.

### Mitochondrial membrane potential

Mitochondrial membrane potential in isolated human platelets (200 × 10^6^ ml^−1^) was measured using a flow cytometer FACSAria III (BD, Franklin Lakes, USA) with Diva version 7.0 acquisition and analysis software, using the probe TMRM (Life Techologies, Ref: T668), in non-quench mode (30 nM)^19^ excited by 561 nM 40 mW laser and collected on 582/15 band pass filter. CD41a-APC (BD Pharmingen, Clone HIP8, Ref: 559777) at 18 times dilution was used to assess platelet activation. Samples were incubated with the probes in MiR05 for 30 min at room temperature. CI was inhibited using 2 μM rotenone. NV189 (250 μM) or DMSO control was added to the samples, followed by oligomycin (1 μg ml^−1^), FCCP (20 μM) and antimycin A (1 μg ml^−1^), the two latter additions as internal controls. Data software used was FlowJo 10 (Tree Star, Ashland, USA). Statistical analyses were performed, and all figures were generated using Prism 6 (GraphPad Software).

### Metabolomics

Isolated human PBMCs (16–25 × 10^6^ ml^−1^) were incubated at 37 °C in 2 ml MiR05 with 5 mM glucose and with rotenone 2 μM or DMSO control. NV189 (250 μM; 0.5 mM total) or DMSO control was added in two subsequent additions. Samples were centrifuged at 4,600*g* for 4 min and the supernatant discarded in two cycles with resuspension of pellet in 1.5 ml of 5% mannitol solution before the second run. To each sample, 800 μl of methanol and 550 μl of solution of the internal standard (H3304-1002, Human Metabolome Technologies Inc., Tsuruoka, Japan) were added and 1 ml of the extracted solution was taken for centrifugation at 2,300*g* at 4 °C for 5 min. Thereafter, 400 μl of the supernatant was filtered at 9,100*g* at 4 °C until no liquid remained. The extract was dried in a centrifugal evaporator (1,500 r.p.m., 1,000 Pa) and put in −80 °C until analysis. Samples were analysed using capillary electrophoresis time-of-flight mass spectrometry (CE-TOFMS) for cationic compounds and capillary electrophoresis tandem mass spectrometry (CE-MS/MS) for anionic compounds (Agilent Technologies, Santa Clara, USA), as previously described^20^. Peaks detected in CE-TOFMS analysis were extracted using automatic integration software (MasterHands ver.2.16.0.15 developed at Keio University) and those in CE-MS/MS analysis were extracted using automatic integration software (MassHunter Quantitative Analysis B.06.00, Agilent Technologies) to obtain peak information including *m/z*, migration time and peak area. The peak area was then converted to relative peak area. The peaks were annotated based on the migration times in CE and *m*/*z* values determined by TOFMS. Putative metabolites were then assigned from Human Metabolome Technologies (HMT) metabolite database on the basis of *m/z* and migration time. All the metabolite concentrations were calculated by normalizing the peak area of each metabolite with respect to the area of the internal standard and using standard curves, which were obtained by three-point calibrations. The lactate:pyruvate ratio was analysed using Friedman's non-parametric paired test for comparison between three groups or more with Dunn's multiple comparisons test of all groups against control. For three data points (one data point in the group treated with only rotenone and two data points in the group treated with rotenone and NV189), pyruvate was below the quantification limit. The estimated lower-quantification limit for pyruvate was between 16.96 and 20.55 pmol per 10^6^ cells and a mean of these two values was used for calculating the lactate:pyruvate ratio for the missing data points. Experiments were performed by the service provider Human Metabolome Technologies Inc. (Tsuruoka, Japan). Cells from the same four healthy volunteers were used for each experimental group.

### Isotope labelling

NV118 was synthesized incorporating all four carbons in the central succinate structure of the molecule with [^13^C] isotopes. Isolated platelets (800 × 10^6^ ml^−1^) were kept at 37 °C in 2 ml MiR05 containing 5 mM glucose. [1, 2, 3, 4-^13^C_4_]NV118 was added in two boluses to a final concentration of 0.5 mM and the samples were incubated for 15, 30, 120 or 240 min. Extracts were prepared as described above. Metabolome measurements were carried out through Human Metabolome Technology Inc., Tsuruoka, Japan. Target metabolites and their isotopomers were annotated based on their theoretical *m/z* value and migration time^21^. Cells from the same two healthy volunteers were used for each experimental group.

### Statistics

Statistical analyses were performed, and all figures generated, using Prism 6 (GraphPad Software, La Jolla, USA) if not otherwise stated. A *P* value of <0.05 was considered statistically significant. No blinding or randomization was performed, except for the metabolomics assays, where the lab performing the analyses was blinded to the intervention allocated to the samples. Data from blood cell respirometry have previously been reported to be normally distributed and parametric tests were used^16^.

### Data availability

All relevant data are contained within the paper and Supplementary Information files or available from the authors upon request.

## Additional information

**How to cite this article:** Ehinger, J. K. *et al*. Cell-permeable succinate prodrugs bypass mitochondrial complex I deficiency. *Nat. Commun.* 7:12317 doi: 10.1038/ncomms12317 (2016).

## Supplementary Material

Supplementary Information Supplementary Figures 1-6 and Supplementary Methods
