# Supplementary material for: Cell-permeable succinate prodrugs bypass mitochondrial complex I deficiency
Source: Nat Commun. 2016 Aug 9;7:12317. doi: 10.1038/ncomms12317 (PMC4980488; doi:10.1038/ncomms12317)
Supplement: Supplementary Information — Supplementary Figures 1-6 and Supplementary Methods [file ncomms12317-s1.pdf]

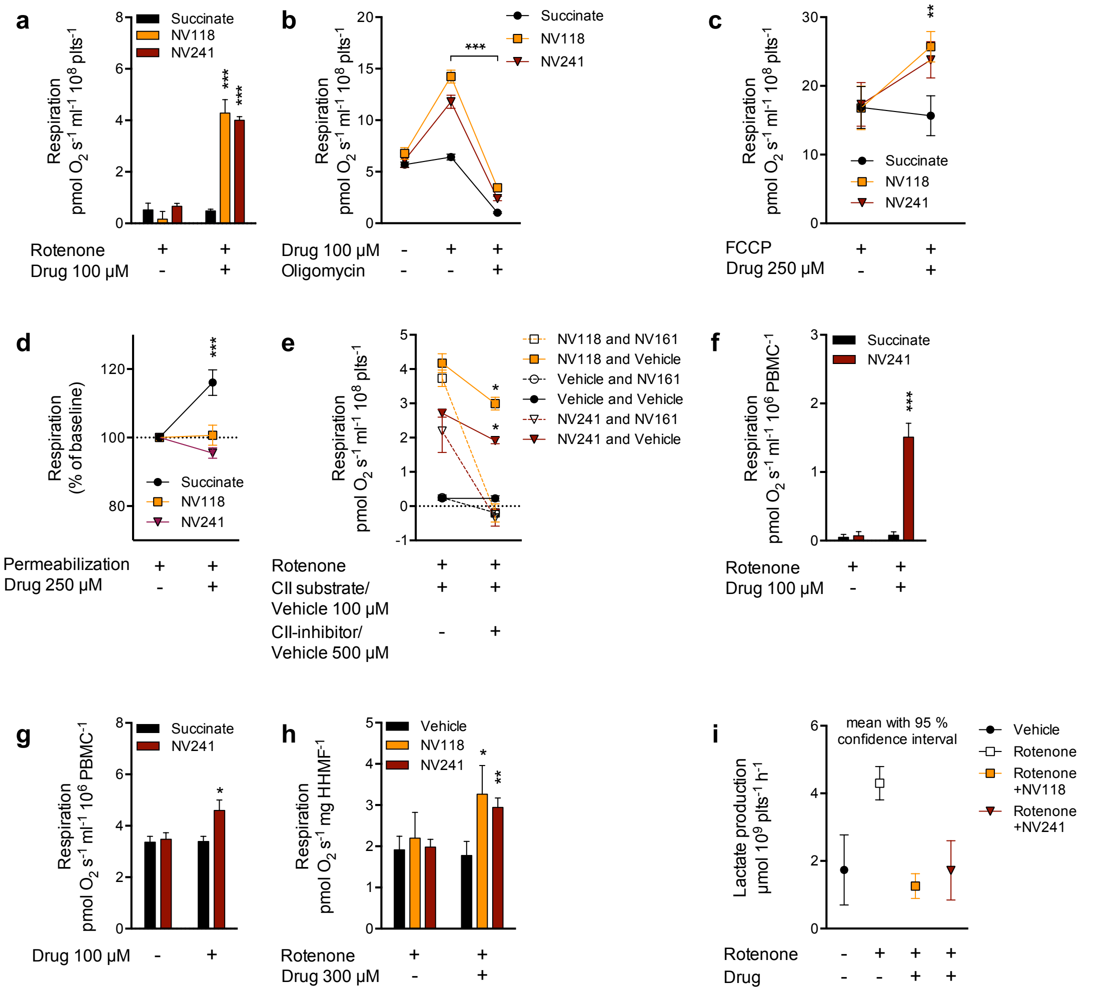

Supplementary Figure 1. Effects of mitochondrial complex II stimulation by succinate prodrugs NV118 and NV241. (**a**) Respiration in platelets (plts) with rotenone-induced mitochondrial complex I (CI) inhibition. (**b**) ATP-generating respiration in platelets, *** indicate significant reduction in drug treated sample pre and post oligomycin. (**c**) Respiration in platelets with FCCP-induced uncoupling. (**d**) Respiration in digitonin-permeabilized platelets. **(e)** Effect on respiration in platelets with addition of the cell-permeable CII inhibitor NV161, * indicate significant difference between NV161 and vehicle for each drug, n=4. (**f**) Respiration in peripheral blood mononuclear cells (PBMCs) with rotenone-induced CI inhibition, n = 4. **(g)** Convergent respiration in PBMCs, n = 4. **(h)** Respiration in human heart muscle fibres (HHMF), n = 5. (**i**) Lactate production in 2 ml buffer containing 400 × 10^6 platelets, incubated with or without rotenone and NV118 or NV241, n = 5. All respirometric experiments in human platelets were performed with n = 6 individuals donors if not otherwise stated. All data presented as mean and standard error if not otherwise stated. In all experiments blood cells from separate donors are used for each n. * = $p < 0.05$, ** = $p < 0.01$, *** = $p < 0.001$ (two-tailed paired or unpaired Student's *t*-test as appropriate, difference between succinate prodrug and control if not otherwise stated).

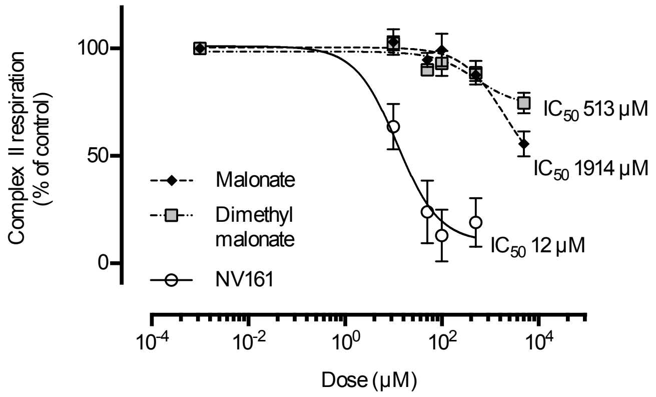

Supplementary Figure 2. Dose-response of three mitochondrial complex II-inhibitors in intact cells. Human platelets (200 × 10^6 ml^{-1}) were incubated as described in the methods section. Rotenone (2 µM) was added to inhibit Complex I and NV241 (100 µM) was added to allow for Complex II-linked respiration. Malonate, dimethyl malonate or NV161 was titrated (from 10 µM to 5 mM final concentration) and rate of oxygen consumption assessed. IC_{50}-values for each compounds was calculated using Prism GraphPad 6.0. NV161 inhibited complex II-supported respiration at lower concentrations than malonate or dimethyl malonate, as depicted by the IC_{50}-values provided in the figure.

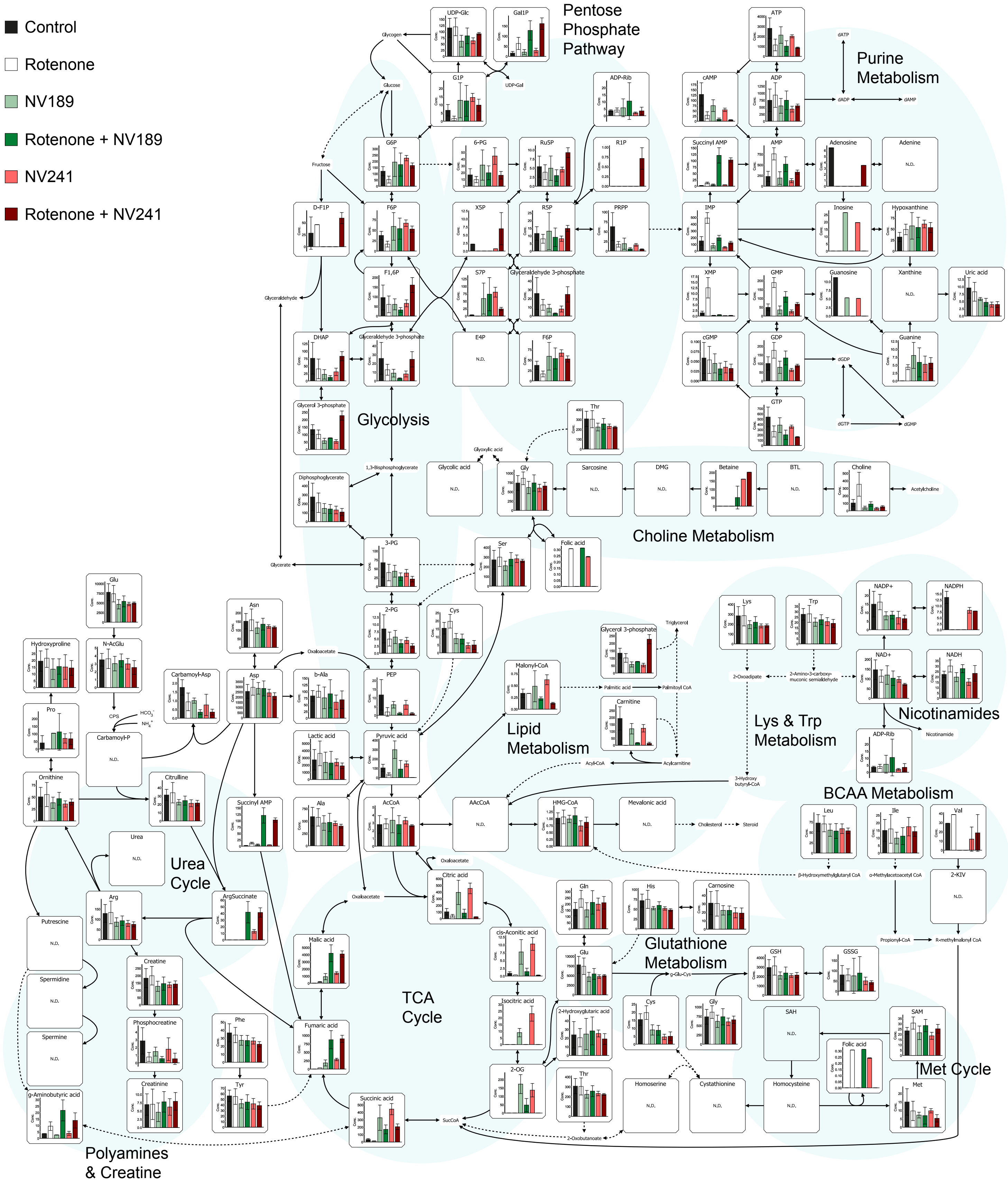

Control
Rotenone
NV189
Rotenone + NV189
NV241
Rotenone + NV241
Pentose Phosphate Pathway
Purine Metabolism
Glycolysis
Choline Metabolism
Lipid Metabolism
Lys & Trp Metabolism
Nicotinamides
BCAA Metabolism
Urea Cycle
TCA Cycle
Glutathione Metabolism
Met Cycle
Polyamines & Creatine
UDP-Glc
Gal1P
Glycogen
Glucose
UDP-Gal
G1P
G6P
6-PG
Ru5P
R1P
ADP-Rib
Fructose
D-F1P
F6P
X5P
R5P
PRPP
F1,6P
S7P
Glyceraldehyde 3-phosphate
Glyceraldehyde
DHAP
E4P
Glycerol 3-phosphate
1,3-Bisphosphoglycerate
Diphosphoglycerate
3-PG
Glycerate
2-PG
PEP
Pyruvic acid
Lactic acid
AcCoA
ATP
ADP
AMP
cAMP
dATP
dADP
dAMP
Succinyl AMP
Adenosine
Adenine
IMP
Inosine
Hypoxanthine
XMP
GMP
Guanosine
Xanthine
Uric acid
cGMP
GDP
GTP
Guanine
dGDP
dGTP
dGMP
Thr
Glyoxylic acid
Glycolic acid
Gly
Sarcosine
DMG
Betaine
BTL
Choline
Acetylcholine
Ser
Folic acid
Cys
N.D.
Glu
N-AcGlu
Hydroxyproline
Pro
CPS
Carbamoyl-P
Carbamoyl-Asp
Asn
Asp
Oxaloacetate
b-Ala
Ala
Ornithine
Citrulline
Urea
Succinyl AMP
ArgSuccinate
Arg
Putrescine
Spermidine
Spermine
g-Aminobutyric acid
Creatine
Phosphocreatine
Creatinine
Phe
Tyr
Malic acid
Fumaric acid
Succinic acid
SucCoA
Citric acid
cis-Aconitic acid
Isocitric acid
2-OG
2-Hydroxyglutaric acid
Malonyl-CoA
Palmitic acid
Palmitoyl CoA
Triglycerol
Carnitine
Acylcarnitine
AcylCoA
AACoA
HMG-CoA
Mevalonic acid
Cholesterol
Steroid
Lys
Trp
2-Oxoadipate
2-Amino-3-carboxy-muconic semialdehyde
3-Hydroxy butyryl-CoA
NADP+
NADPH
NAD+
NADH
Nicotinamide
ADP-Rib
Leu
Ile
Val
β-Hydroxymethylglutaryl CoA
α-Methylacetoacetyl CoA
2-KIV
Propionyl-CoA
R-methylmalonyl CoA
Gln
His
Carnosine
GSH
GSSG
g-Glu-Cys
Homoserine
Cystathionine
Homocysteine
SAH
SAM
Met
2-Oxobutanoate
Conc.

Supplementary Figure 3. Intracellular metabolism of exogenous succinate. Overview of metabolic pathways in peripheral blood mononuclear cells incubated with or without rotenone (2 µM) and with or without NV189 or NV241. Metabolites were quantified using capillary electrophoresis time-of-flight mass spectrometry for cationic compounds and capillary electrophoresis tandem mass spectrometry for anionic compounds. Delivery of intracellular succinate and anaplerosis of TCA cycle intermediates were confirmed. The branched-chain amino acids valine (Val) and isoleucine (Ile) can be metabolized to succinyl-CoA but are primarily metabolized in muscle tissue. Valine was only detected in very few samples and altogether these data were inconclusive. Total succinyl CoA-related amino acids revealed no difference between the treatment groups. The levels of reduced glutathione (GSH) in samples treated with NV189 or NV241 were marginally decreased, but the glutathione redox ratio was unaltered. Cysteine (Cys), a precursor to glutathione, which in itself also holds antioxidant properties, was decreased in samples treated with NV189 or NV241. Altogether the data cannot exclude oxidative stress. ATP levels were decreased similarly in all rotenone-treated samples. ADP levels changed in the opposite direction, and thus the interrelated adenylate energy charge clearly indicated aggravated energy status of the rotenone-treated cells. Guanylate energy charge also indicates the energy status and displays equivalent results. Furthermore, NADH was increased in all samples treated with rotenone. Creatine levels, also linked to energy status of the cell, though primarily in muscle, did neither differ significantly. Overall the drug treated samples did not display a significant change in the energy status of the cells as measured by metabolomics. The data related to glycolytic flux is not conclusive. Glucose 6-phosphate (G6P) displayed somewhat higher values in the drug-treated samples, as did fructose 6-phosphate (F6P). All data presented as mean and SD using cells from same 4 individuals for each treatment group.

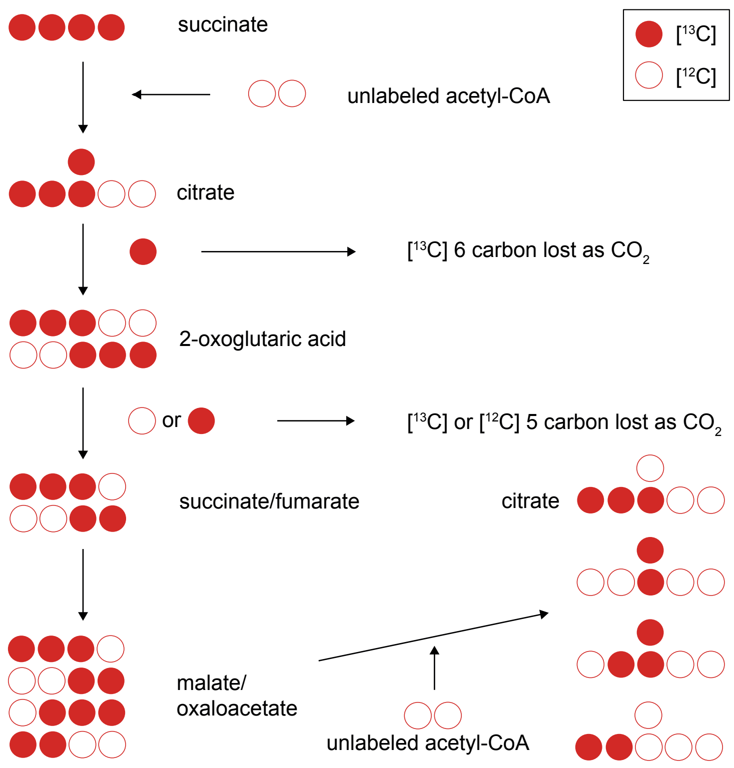

Supplementary Figure 4. Metabolic route of isotope labeled compounds. Putative metabolic incorporation of carbons from [1,2,3,4-$^{13}C_4$]NV118 through the TCA cycle. In order to achieve [$^{13}C_6$]citrate, [$^{13}C_2$]acetyl-CoA must be generated.

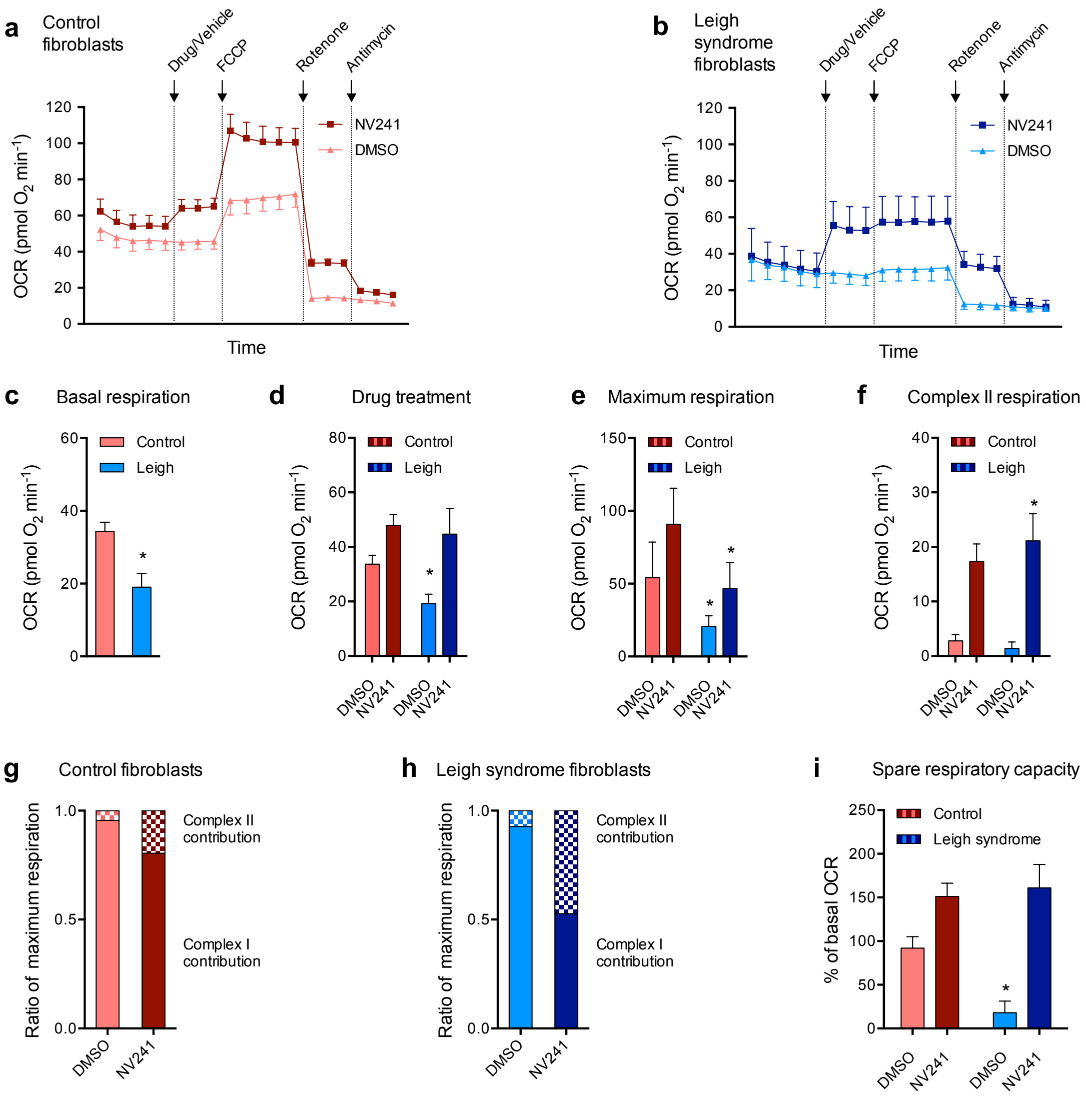

Supplementary Figure 5. Treatment of mitochondrial complex I-deficient Leigh syndrome patient fibroblasts with NV241. (**a-b**) Oxygen consumption rate (OCR) in three control fibroblast lines and a mitochondrial complex I-deficient cell line (recessive *NDUFS2* mutation) treated with NV241 or vehicle. (**c-f**) Quantification of OCR in control and patient fibroblasts for each respiratory state. (**g-h**) Relative contribution of CII (and CI)-linked respiration to maximum uncoupled respiration in patient cells and control cell lines (4.4 % in the control cell lines and 7.3 % in the Leigh syndrome cells; with treatment, this increased to 19.6 % in control cells, and to 47.3 % in patient cells). (**i**) Spare respiratory capacity, defined as percent increase from endogenous baseline to maximum uncoupled respiration. Data presented as mean and standard error of n = 3 separate experiments performed with eight technical replicates for each cell lines. Data from the three control cell lines are pooled. * = $p < 0.05$ (two-tailed unpaired Student's *t*-test, difference between Leigh and control cell lines).

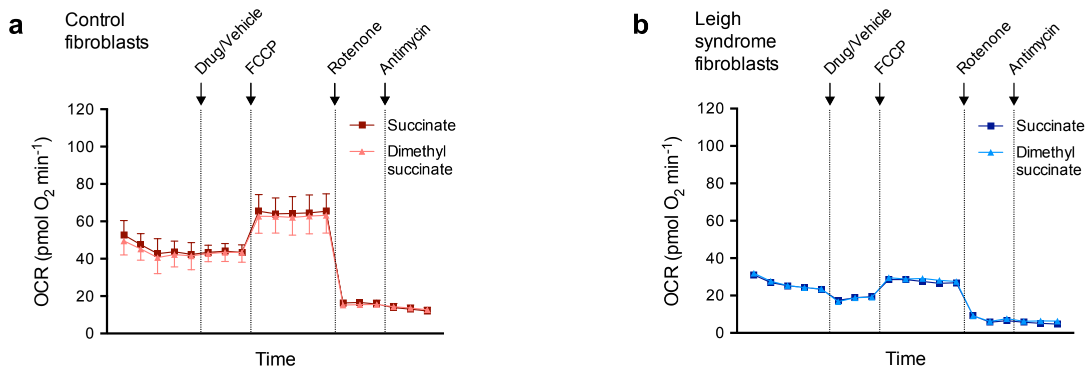

Supplementary Figure 6. Effects of succinate and dimethyl succinate on complex I-deficient Leigh syndrome patient fibroblasts. Oxygen consumption rate (OCR) in three control fibroblast lines (**a**) and a mitochondrial complex I-deficient cell line (**b**) (recessive *NDUFS2* mutation) treated with succinate or dimethyl succinate. Each experiment performed with eight technical replicates and n = 1 for each cell line. Data from the three control cell lines are pooled.

Supplementary Methods

Succinic acid diacetoxymethyl ester (NV101-118)

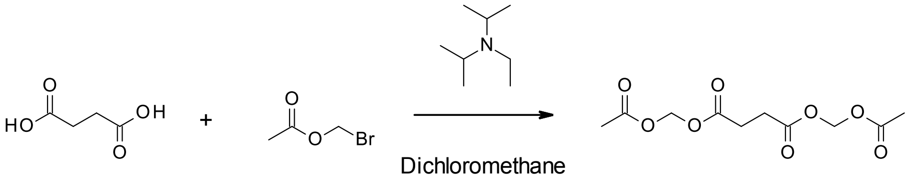

Succinic acid (58.6 g, 0.496 mol) was added to dichloromethane (2 L) and the mixture cooled to 0°C. Diisopropylethylamine (201 mL, 1.154 mol) was added during 20 minutes followed by bromomethyl acetate (159.4 g, 1.042 mol) during 30 minutes and the solution stirred overnight under an atmosphere of nitrogen. The solution was cooled to 0°C and washed successively with 1 L of cold 1% hydrochloric acid, 0.6% hydrochloric acid and water (x3). The solution was treated with decolourizing charcoal, dried with magnesium sulphate and concentrated to an oil which was crystallized from diethyl ether (200 mL)/*iso*hexane (10 mL) to afford 92 g of succinic acid diacetoxymethyl ester as a white solid. m.p.: 59–60°C.

^{1}H NMR (300 MHz, $CDCl_3$, ppm) δ 5.76 (s, 4H), 2.72 (s, 4H), 2.13 (s, 6H). ^{13}C NMR (100 MHz, $CDCl_3$, ppm) δ 170.8, 169.6, 79.2, 28.5, 20.7. LCMS (m/z) 263.2 $[M+H]^+$, 285.1 $[M+Na]^+$.A further 8 g of pure material was obtained from concentration of the liquors.

Succinic acid bis-(1-acetoxy-ethyl) ester (NV101-189)

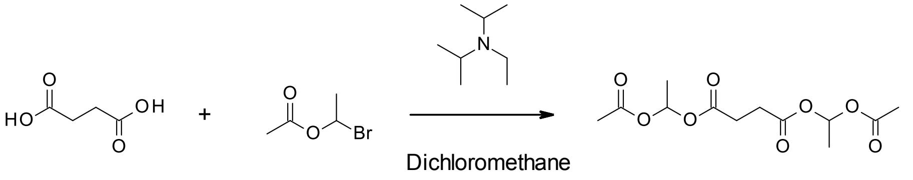

Succinic acid (58.6 g, 0.496 mol) was added to dichloromethane (2 L) and the mixture cooled to 0°C. Diisopropylethylamine (201 mL, 1.154 mol) was added during 20 minutes followed by 1-bromoethyl acetate (175 g, 1.05 mol) during 30 minutes and the solution stirred overnight under an atmosphere of nitrogen. The solution was cooled to 0°C and washed successively with cold (1.5 L quantities) of water, 1% hydrochloric acid (twice), sodium bicarbonate solution and water. The solution was dried with magnesium sulphate and concentrated to an oil which was crystallized from *t*-butylmethyl ether to afford 41 g of succinic acid bis-(1-acetoxy-ethyl) ester as a white solid. m.p.: 63–64°C.

^{1}H NMR (300 MHz, $CDCl_3$, ppm) δ 6.87 (q, J=5.5 Hz, 2H), 2.66 (m, 4H), 2.07 (s, 6H), 1.48 (d, J=5.4 Hz, 6H). ^{13}C NMR (100 MHz, $CDCl_3$, ppm) δ 170.5, 169.3, 89.1, 29.1, 21.4, 20.0. LCMS (m/z) 313.0 $[M+Na]^+$

Succinic acid 1-acetoxy-ethyl ester acetoxymethyl ester (NV101-241)

i) Succinic acid 1-acetoxy-ethyl ester *tert*-butyl ester

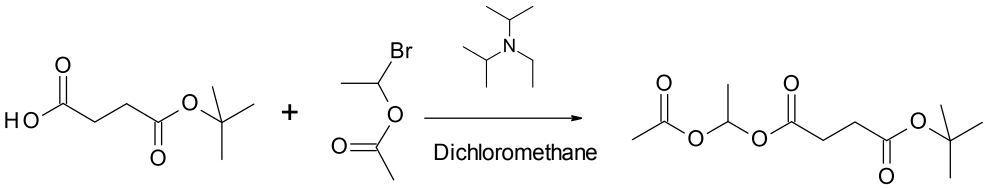

To a solution of succinic acid mono-*tert*-butyl ester (136.15 g, 781 mmol) in dichloromethane (1 L) at -5°C was added di-isopropylethylamine (163.36 mL, 121.21 g, 938 mmol, 1.2 eq). Acetic acid 1-bromo-ethyl ester (143.57 g, 860 mmol, 1.1 eq) was then added dropwise. The reaction was then stirred for 16 hours at room temperature. The reaction was concentrated *in vacuo*, and the residue was dissolved in EtOAc (500 mL) and this was washed sequentially with 0.5 M aq HCl (2 x 450 mL), saturated aq $NaHCO_3$ (500 mL), and brine (400 mL). The organics were then dried over $MgSO_4$. The residue was purified by vacuum distillation (vapour temperature 109-115°C, pressure <20 mbar) to give the title compound as a colourless oil (140.5 g, 539.8 mmol, 69%).
^{1}H NMR (300 MHz, $CDCl_3$, ppm) δ 6.88 (q, J=5.5 Hz, 1H), 2.50-2.65 (m, 4H), 2.07 (s, 3H), 1.48 (d, J=5.5 Hz, 3H), 1.45 (s, 9H).

ii) Succinic acid mono-(1-acetoxy-ethyl) ester

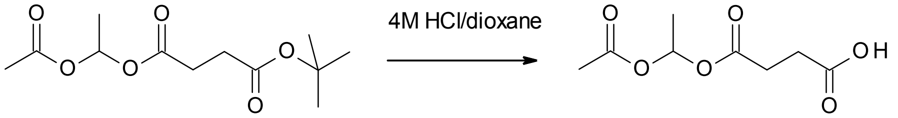

Succinic acid 1-acetoxy-ethyl ester *tert*-butyl ester (74.8 g, 288 mmol) was dissolved in 4 M HCl in dioxane (720 mL, 2.88 mol, 10 eq), and the reaction was stirred for 5 hours at 40°C. The reaction was concentrated *in vacuo*, and the residue was azeotroped with toluene (500 mL), to give the desired product as a clear oil (58 g).
^{1}H NMR (300 MHz, $CDCl_3$, ppm) δ 10-14 (br, 1H), 6.73 (q, J=5.5 Hz, 1H), 2.40-2.60 (m, 4H), 2.03 (s, 3H), 1.40 (d, J=5.5 Hz, 3H).

iii) Succinic acid 1-acetoxy-ethyl ester acetoxymethyl ester (NV101-241)

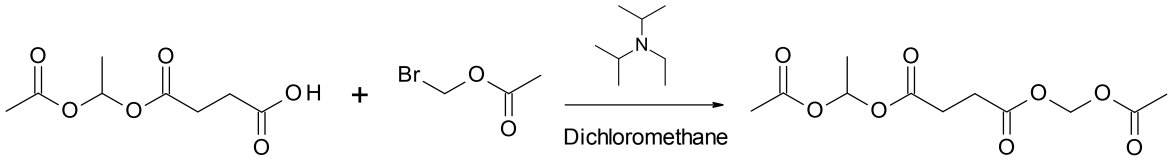

To a solution of succinic acid mono-(1-acetoxy-ethyl) ester (58.7 g, 287 mmol) in dichloromethane (600 mL) at -5°C was added di-isopropylethylamine (60.1 mL, 44.6 g, 345 mmol, 1.2 eq). Acetic acid 1-bromo-methyl ester (31.0 mL, 48.36 g, 316 mmol, 1.1 eq) was then added dropwise. The reaction was then stirred for 4 hours at room temperature. The reaction was concentrated in vacuo, and the residue was dissolved in EtOAc (450 mL) and this was washed sequentially with cold (5°C) 0.5 M aq HCl (400 mL), saturated aq $NaHCO_3$ (400 mL), and brine (350 mL). The organics were then dried over $MgSO_4$. The residue was purified by vacuum distillation (vapour temperature 154-169°C, pressure 3.2 mbar) to give the title compound as a pale yellow oil (57.45 g, 208 mmol).

^{1}H NMR (300 MHz, $CDCl_3$, ppm) δ 6.87 (q, J=5.5 Hz, 1H), 5.76 (s, 2H), 2.60-2.77 (m, 4H), 2.13 (s, 3H), 2.08 (s, 3H), 1.49 (d, J=5.5 Hz, 3H). ^{13}C NMR (100 MHz, $CDCl_3$, ppm) δ 170.9, 170.0, 169.6, 168.9, 88.7, 79.2, 28.6, 20.8, 20.7, 19.4. LCMS (m/z) 277.0 $[M+H]^+$, 299 $[M+Na]^+$.

Malonic acid diacetoxymethyl ester (NV01-161)

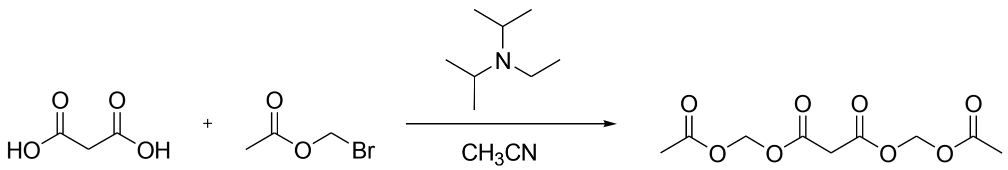

To a mixture of malonic acid (0.30 g, 2.9 mmol) and di-isopropylethylamine (1.50 g, 11.5 mmol) in acetonitrile (5 mL) was added bromomethyl acetate (0.96 g, 6.4 mmol). The reaction was stirred at room temperature overnight. After removing the solvent, the residue was purified by silica gel chromatography (eluting with petroleum ether and ethyl acetate (9/1 to 3/1)) to give the title compound as a colourless oil (119 mg, 17% yield).

^{1}H NMR (300 MHz, $CDCl_3$, ppm) δ 5.78 (s, 4H), 3.50 (s, 2H), 2.14 (s, 6H). ^{13}C NMR (100 MHz, $CDCl_3$, ppm) δ 169.4, 164.6, 79.5, 40.8, 20.6. LCMS (m/z) 266.2 $[M+NH_4]^+$, 271.1 $[M+Na]^+$.
